# *Trans-*Cultural Validation of the “Academic Flow Scale” (Flow 4D 16) in Arabic Language: Insights for Occupational and Educational Psychology From an Exploratory Study

**DOI:** 10.3389/fpsyg.2019.02330

**Published:** 2019-10-15

**Authors:** Nasr Chalghaf, Chiraz Azaiez, Hela Krakdiya, Noomen Guelmami, Tania Simona Re, Juan José Maldonado Briegas, Riccardo Zerbetto, Giovanni Del Puente, Sergio Garbarino, Nicola Luigi Bragazzi, Fairouz Azaiez

**Affiliations:** ^1^Department of Health Sciences (DISSAL), Postgraduate School of Public Health, University of Genoa, Genoa, Italy; ^2^Group for the Study of Development and Social Environment (GEDES), Faculty of Human and Social Science of Tunis, Tunis, Tunisia; ^3^Higher Institute of Sport and Physical Education of Sfax, University of Sfax, Sfax, Tunisia; ^4^Higher Institute of Sport and Physical Education of Kef, University of Jendouba, Jendouba, Tunisia; ^5^Research Unit, Sportive Performance and Physical Rehabilitation, High Institute of Sports and Physical Education of Kef, University of Jendouba, Jendouba, Tunisia; ^6^UNESCO Chair “Health Anthropology, Biosphere and Healing Systems”, University of Genoa, Genoa, Italy; ^7^Department of Psychology and Sociology of Education, University of Extremadura, Badajoz, Spain; ^8^Centro Studi Terapia della Gestalt, Milan, Italy; ^9^Department of Neuroscience, Rehabilitation, Ophthalmology, Genetics, Maternal and Child Health (DINOGMI), University of Genoa, Genoa, Italy; ^10^Laboratory for Industrial and Applied Mathematics, Department of Mathematics and Statistics, York University, Toronto, ON, Canada

**Keywords:** *trans-*cultural validation of a scale, Arabic language, academic flow, occupational psychology, students

## Abstract

**Background:** As an optimal psychological state, flow represents those moments when everything comes together for the performer. Flow is often associated with high levels of performance and is a positive psychological experience.

**Aim:** Our study aimed to validate the “Academic Flow Scale” (Flow 4D 16) in Arabic language across Tunisian population, and to test its factor structure, in terms of internal consistency/reliability, predictive validity, and sensitivity.

**Methods:** The population is composed of 320 students (139 men and 181 women) belonging to the University of Sfax, with a mean age of 22.26 years. The students voluntarily responded to the scale of academic flow (Flow 4D 16). Both exploratory (EFA) and confirmatory (CFA) factor analyses were performed.

**Results:** The four-dimensional alpha coefficients of the Flow 4D 16 indicate an excellent internal consistency, respectively, of 0.902 (Cognitive), 0.959 (Time), 0.974 (Ego) and 0.960 (Well-being). The CFA fit indices were satisfactory.

**Conclusion:** In summary, the 16-items model (original version) showed for all the indices an excellent fit to the theoretical model, confirming the four-dimensional factor structure among Tunisian student population.

## Introduction

The concept of “flow” or “psychological flow” can be defined as “the optimal experience” that a subject can make. It generally occurs when an individual is engaged in a specific activity with clear goals and high commitment, facing challenges in proportion to his/her skills, fully mobilizing his/her competencies, and dedicating his/her attention to the task. Indeed, flow occurs when the subject is totally absorbed by what is doing and becomes unconscious of himself/herself. In this case, he/she forgets the time that passes, as well as other potential sources of distraction, even including his/her bodily needs.

The Hungarian psychologist Mihály Csíkszentmihályi has developed the concept of “flow”, being considered the founding father and the pioneer of the flow studies. His works published between 1975 and 2000 aimed to study the context of appearance and emergence of the optimal experience peak and the structure of pleasure generated. His investigations are generally based on the descriptions of the experiences of individuals who feel this pleasure in the practice of their activity where the intrinsic reward is considered essential (Nakamura and Csikszentmihalyi, 2002).

There are a lot of flow studies, among which we can take into particular consideration the classification of some scholars (Walker, 2010; Borderie and Michinov, 2014; Borderie, 2015) who distinguish between two types of flow.

The first approach is represented by the model of individual flow: the group to which the individual belongs is considered only as the context of the emergence of the state of flow. The collective entity to which the subject belongs can have just a minimal effect on his/her state.

The second model is characterized by the concept of group flow that corresponds to the collective optimal experience. This takes place when individuals act in co-presence (Johnson and Johnson, 1989; Walker, 2010; Borderie and Michinov, 2014; Borderie, 2015).

Other researchers (Fong et al., 2014) have re-examined, in a comparative analysis, what Csikszentmihalyi (2004) has called “the paradox of work”: that is to say, the fact that people experience the flow more often at workplace than during their leisure time. The results found by their meta-analysis do not differ significantly from those obtained for a very short period (only 7 days) among 100 workers working in different occupational contexts and jobs. This could be explained by taking into account the value given to leisure, which is higher so that people tend to prefer leisure, which also correspond to periods of less stress and activation (Engeser and Baumann, 2016).

Many scholarly researches as well as practical applications have taken place after the introduction of the seminal concept of optimal experience. Flow appears particularly in the school world, impacting on different variables and constructs, from well-being to performance, academic achievements, career expectations and future occupational employment (Cortini et al., 2010). Indeed, Csikszentmihalyi and Rathunde (1993), in a longitudinal survey of more than 200 adolescents, showed that the student’s optimal feeling at school was the best predictor of his/her subjective engagement and how he/she fully used his/her potential. Larson (2011) has shown how flow can be exploited in order to improve school learning at a higher level, emphasizing the main conditions of the optimal experience including the autonomy, interest and commitment of the learners. Much more, the study of Carpentier et al. (2012) showed that subjects who have an intrinsic and self-controlled motivation, under the influence of a “harmonious passion”, can have a greater tendency to live the experience of flow in their favorite activities.

In this context, the relation between flow and positive affect often appears in undergraduate students (Rogatko, 2009). Furthermore, Kulkarni et al. (2015) conducted three experiments with undergraduate university students using a simple and interesting on-screen game. They manipulated the flow level by including more or less flow ingredients. They showed that the presence of the main antecedents (commitment, feedback and balance-skill) produced a higher level of flow (pleasure, interest, concentration) and decreased the defensive behaviors.

Given the importance of the concept of flow in educational contexts and milieus, here briefly overviewed, and given that no instrument exists in Arabic language, the aim of the present study was to provide a *trans-*cultural validation of the “Academic Flow Scale” (Flow 4D 16).

## Materials and Methods

### Participants

The population under study was made up of 320 students (139 men and 181 women) (Table 1) belonging to different study sections (beauty arts, law, humanities, economics, sports and physical education, and medicine) of the University of Sfax, Sfax, Tunisia, with a mean age of 22.26 ± 1.86 years. All participants in our study were volunteers, taking part into the study in an anonymous and confidential way. They gave their written, informed consent to be part of the investigation and were carefully and extensively advised about the aim of the study. They were free to withdraw their participation at any moment of the investigation. The study protocol was in-depth reviewed and received the full approval by the ethical committee of the University of Sfax, Sfax, Tunisia.

**TABLE 1:** Distribution of the study population by sex and study sections.

**Sex**	**Study sections**						**Total**
	**Beauty arts**	**Law**	**Humanities**	**Economics**	**Sports and physical education**	**Medicine**	
Male	40	37	26	21	9	6	139
	12.5%	11.6%	8.1%	6.6%	2.8%	1.9%	43.5%
Female	58	65	23	17	16	2	181
	18.1%	20.3%	7.2%	5.3%	5.0%	0.6%	56.5%
Total	98	102	49	38	25	8	320
	30.6%	31.9%	15.3%	11.9%	7.8%	2.5%	100.0%

### Measurement

Several questionnaires exist to measure the flow. On the one hand, the Mayers, 1978 Flow Scale a self-descriptive measuring instrument, enables scholars to estimate how often a person experiences each of the nine dimensions of flow. This tool can be used for repeated measurements (Delle Fave and Massimini, 1988) in order to evaluate the differences in the state of flow according to the activity practiced at various moments. On the other hand, more recently, a flow scale has been developed, based on questionnaires developed in specific contexts such as sports and physical education (Jackson and Marsh, 1996; Jackson and Eklund, 2002) and psychology/psychotherapy (Demontrond and Gaudreau, 2008). Within this field, there exists a French version of the Flow State Scale (FSS) composed of 32 items divided into four dimensions (Jackson and Eklund, 2002; Fournier et al., 2007).

Furthermore, there exists a French version of the Flow scale adapted to specifically study the engagement and involvement of students in collective work, and especially how they live the optimal experience (flow) within an educational context. This measuring instrument of the optimal experience (Flow 4D 16) in an educational context was developed by Heutte and Fenouillet (2010). This tool contains four dimensions (each one is consisting of four items) on a 7-degree Likert scale ranging from 1 (“strongly disagree”) to 4 (“indifferent”) and 7 (“strongly agree”).

In our study, we have chosen this scale to assess the academic flow in a sample of Tunisian students.

### Procedure

Our procedures for translating and adapting the flow scale (4D 16) validated in French by Heutte and Fenouillet (2010) were based on two subsequent translation steps (namely, forward translation and back translation) of this measurement tool, from the original language to the target language (Arabic). This allows the detection of divergent errors and interpretations of certain ambiguous items of the original version during initial translations. Then, we made a back-translation to the French language from versions translated into Arabic.

The translations were done by two teams of translators. Each team was made up of two translators. Their characteristics and personal qualifications are important in terms of knowledge of both the source language and of the target language.

### Statistical Analysis

Data normality was checked assessing the skewness and kurtosis. To verify the psychometric quality of the construct, the internal consistency was assessed computing the Cronbach’s alpha coefficient. Temporal stability of the questionnaire was calculated (test-retest). Predictive validity was tested by calculating the correlation matrix and the Pearson correlation coefficient.

To investigate the factor structure of the questionnaire, an orthogonal *Varimax* type exploratory factor analysis (EFA) (Kaiser, 1958) with a principal-component analysis (PCA) was performed on our questionnaire from the 16 items of the tool (Jackson and Marsh, 1996). The item was retained if the loading was satisfactory, there is to say equal to or greater than 0.40 (Archer et al., 1997). The sampling adequacy was measured computing the Kaiser-Meyer-Olkin (KMO).

A confirmatory factor analysis (CFA) of the first order with maximum likelihood estimation made it possible to verify the factorial structure in four dimensions. In order to test the adequacy of the collected data to the theoretical model, it is generally recommended to use several types of indices (Schermelleh-Engel et al., 2003). Thus Roussel et al. (2002) advise to present at least two indices of adjustment *per* family of indices. These indices make it possible to evaluate to what extent the theoretical model posited *a priori* correctly reproduces the data. The most common index is the Chi-square (Satorra and Bentler, 1994), which should be not statistically significant. For a further assessment of the degree of fit of the model, we used the Goodness of Fit Index (GFI) (Marsh et al., 1988; Jöreskog and Sörbom, 1996a, b; Schermelleh-Engel et al., 2003), as well as its adjusted value (AGFI), which should be equal to or greater than 0.90 and 0.85, respectively (Schermelleh-Engel et al., 2003). Then we relied on the Root Mean Square Error of Approximation (RMSEA). This index, differently from the GFI, tests the wrong adjustment. It should be less than 0.05 and 0.08 for a good and acceptable fit, respectively, according to some scholars (Jöreskog and Sörbom, 1996a, b; MacCallum et al., 1996), who also suggest to use the ratio between the Chi-square and the number of degrees of freedom in order to distinguish between the “over-adjusted” and “under-adjusted” models. The target threshold generally proposed by Carvalho and Chima (2014) is ≤3. However, some authors (Roussel et al., 2002) agree on an acceptance threshold of ≤2. Furthermore, we utilized also the standardized root mean square residual (SRMR), which should be ≤0.10 for an acceptable fit (Schermelleh-Engel et al., 2003). Moreover, the Comparative Fit Index (CFI), the Not Normed Fit Index (NNFI), the Normed Fit Index (NFI) and the Parsimony NFI (PNFI) are also particularly relevant especially when it comes to comparing different alternative models. NFI value should be ≥0.90, CFI, NFI, NNFI, and PNFI values should be ≥0.95 (Byrne, 1998; Schermelleh-Engel et al., 2003).

Further, a sensitivity analysis (analysis of variance, ANOVA) was done to assess the impact of age, gender and kind of study on the scores of each dimension of the questionnaire.

Exploratory factor analysis was conducted on a random split-half sample, whereas CFA was performed on the other split-half sample. EFA was carried out by means of the commercial software “Statistical Package for Social Sciences (SPSS for Windows, version 24, IBM, Armonk, NY, United States), whereas CFA was conducted with AMOS (version 24, IBM, Armonk, NY, United States).

## Results

### Quality of the Construct

Data were normally distributed in terms of skewness and kurtosis. Furthermore, our results indicated that the scale of students’ academic flow (Heutte and Fenouillet, 2010) had a good temporal stability (*r* = test and re-test = 0.886).

Predictive validity was tested by calculating the Pearson correlation. The results obtained from the correlation matrix between the 16 statements of the Academic Flow indicated that there was a positive correlation at *p* < 0.001 between most variables. The coefficient r was between 0.110 and 0.916. For some statements, correlations were found to be good, such as the correlation between Item 12 (“I am not concerned about what others might think of me”) and Item 10 (“I’m not concerned about the judgment of others”) (*r* = 0.916 at *p* < 0.01), as well as the correlation between Item 12 (“I am not concerned about what others might think of me”) and item 9 (“I do not care what others may think of me”) (*r* = 0.911 at *p* < 0.01). However, there are items inversely correlated, such as the correlation between Item 2 (“I feel that I control my actions perfectly”) and Item 8 (“(“It seems to me that time passes slowly or quickly”) (*r* = –0.135 at *p* < 0.05), However, there are some coefficients that are small, for example the coefficient of the correlation between Item 3 (“At each step, I know what I have to do”) and Item 12 (“I am not concerned about what others might think of me”) (*r* = 0.110 at *p* < 0.05), as well as the coefficient of the correlation between Item 2 (“I feel that I control my actions perfectly”) and Item 15 (“This activity is highly entertaining for me”) (*r* = 0.114 at *p* < 0.05).

### Exploratory Factor Analysis of the Academic Flow Scale

The results of the EFA showed that the students’ academic flow scale well reproduced the expected theoretical model (in terms of homogeneity of items) with an overall good internal consistency (α = 0.886). Each dimension (Table 2) had an excellent internal consistency, respectively, of 0.902 (Cognitive), 0.959 (Time), 0.974 (Ego), and 0.960 (Well-being). The KMO indicated a good sampling adequacy (KMO = 0.842 at *p* < 0.001). The eigenvalue of the dimension of altered perception of time was 4.23, which corresponded to 26.47% of the total variance whereas the eigenvalue of the dimension of well-being was 3.45, explaining up to 48.07% of the total variance. The eigenvalue of the dimension of dilation of ego was 3.38 explaining up to 69.20% of the total variance. Finally the eigenvalue of the dimension of cognitive absorption was 2.89, explaining up to 87. 29% of the total variance.

**TABLE 2:** Factor structure of the scale of flow in Arabic language.

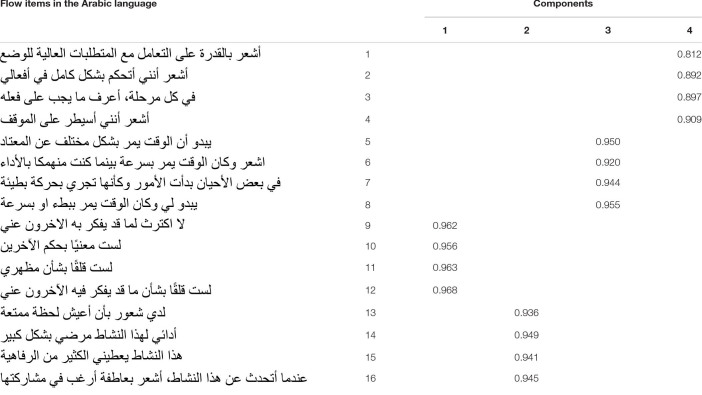

*(English translation of the items). Item 1. I feel that I am able to deal with the high requirements of the situation. Item 2. I feel that I control my actions perfectly. Item 3. At each step, I know what I have to do. Item 4. I feel like I can control the situation. Item 5. Sometimes time seems to pass differently than usually. Item 6. I feel that time is running fast when I am busy. Item 7. Sometimes things seem to start to run like a slow motion. Item 8. It seems to me that time passes slowly or quickly. Item 9. I do not care what others may think of me. Item 10. I’m not concerned about the judgment of others. Item 11. I am not worried about my appearance. Item 12. I am not concerned about what others might think of me. Item 13. I have a feeling that I am living a pleasant situation. Item 14. My performance of this activity is highly satisfactory. Item 15. This activity is highly entertaining for me. Item 16. When I talk about this activity, I feel a passion that I would like to share.*

### Confirmatory Factor Analysis of the Flow Scale

Our model had a statistically significant Chi-square [χ^2^ = 121.542, 96 degrees of freedom at *p* < 0.05]. The GFI (0.956) was satisfactory, the NFI was 0.979, the NNFI was 0.994. Furthermore, the CFI was 0.996, the AGFI was 0.938, the RMSEA was 0.029, the SRMR was 0.007, and the PNFI was 0.994.

**FIGURE 1:**
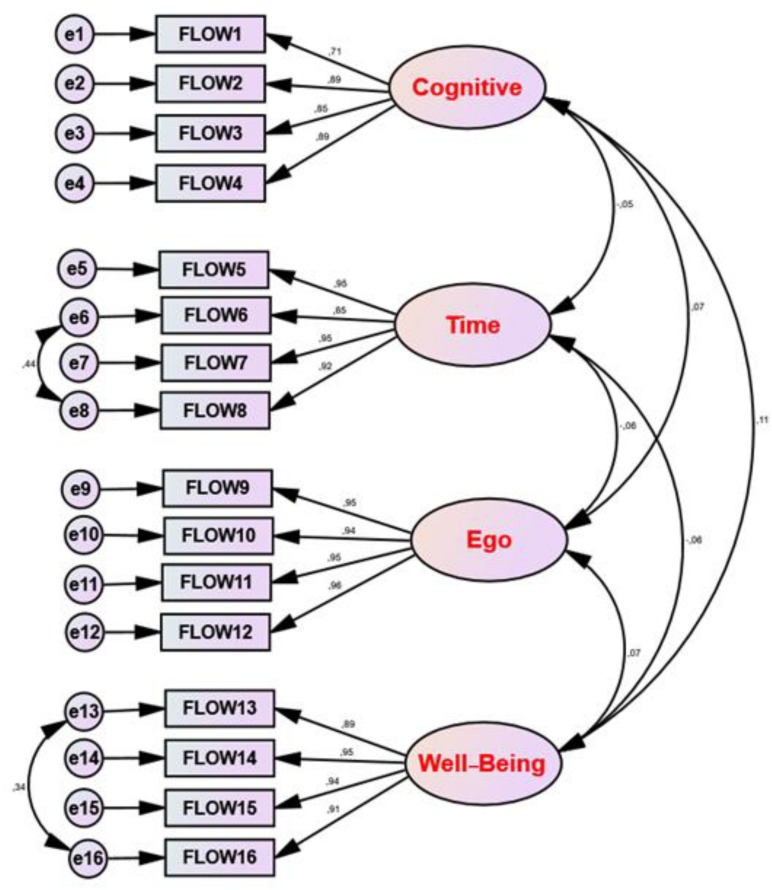
Standardized results of the confirmatory factor analysis (CFA) of academic flow scale (4D 16) in Arabic version.

In summary, the 16-item model showed for all the indices tested an excellent fit to the theoretical model, confirming the four-dimensional factor structure for the Tunisian student population (Figure 1).

### Sensitivity Analysis

From the findings of the ANOVA, the sensitivity analysis showed that women had a more altered perception of time with respect to men. There was also an age effect on cognitive absorption, whereas kind of study impacted on all domains except for time. On the other hand, in terms of effects of interaction, no significant influence could be detected (Table 3).

**TABLE 3:** Impact of sex, age, kind of study and their interaction effects on the dimensions of the Flow Scale (4D 16).

**Variables**	**F**			
	**Cognitive**	**Time**	**Ego**	**Well being**
Gender	1.458	15.051**^∗∗^**	0.502	0.358
Age	2.432**^∗^**	0.347	1.591	1.615
Kind of study	2.304**^∗^**	0.437	2.308**^∗^**	2.348**^∗^**
Gender X age	0.816	1.924	1.534	1.475
Gender X kind of study	0.318	1.022	0.769	0.699
Age X kind of study	1.332	0.509	0.758	0.907
Gender X Age X kind of study	1.383	1.161	1.536	1.616

*^∗^Statistically significant at *p* < 0.05, ^∗∗^Statistically significant at *p* < 0.01.*

## Discussion

The flow as it was conceived by Seligman and Csikszentmihalyi (2000) as a state of deep absorption by the task and intense concentration of the subject was later conceptualized as a more complex state, a broader provision, and a domain-specific disposition (Jackson et al., 2008). Over the last two decades, researchers in the field of occupational psychology have increasingly focused on the emergence of flow in the context of work in a wide range of occupations and organizational contexts, including scientists (Quinn, 2005), and teachers (Salanova et al., 2006).

In this context, the researchers have investigated the flow at work and its determinants, including individual differences (Eisenberger et al., 2005), factors related to the working environment (Mäkikangas et al., 2010), as well as the consequences of flow at workplace, such as significant improvements in the psychological well-being of employees (Debus et al., 2014), their performance and achievements.

Despite the richness of these studies on the academic flow, we noticed an absence of work on flow among Arabic-speaking students, which has encouraged us to perform a *trans-*cultural validation of the scale of the academic flow of Heutte and Fenouillet (2010) in Arabic language.

Our results suggested that the Arabic version of the academic flow scale is a valid and reliable scale for assessing flow among students from different academic sections and both sexes. The results here presented indicated that the scale of assessment of students’ academic flow (Heutte and Fenouillet, 2010) had a good internal consistency and temporal stability. These results corroborated those found by other scholars (Karageorghis et al., 2000; Jackson and Eklund, 2002; Jackson et al., 2008).

In addition, the results of the EFA showed that this scale well reproduced the expected theoretical model (in terms of homogeneity of the items) with an interesting internal consistency for each dimension extracted.

Furthermore, the results of the CFA showed an excellent fit to the theoretical model, confirming in a satisfactory way the four-dimensional factor structure in a population of Tunisian university students. However, the CFA presented also some error correlations (namely, between e6 and e8, and between e13 and e16). This could be explained taking into account items formulation and, mainly, the overlap in their content.

However, our study is not without limitations. The Arabic-speaking world is quite vast and culturally different, therefore limiting the investigation to Tunisian subjects could influence the general extensibility of the results. Furthermore, only a sample of students from the same university was studied, even though from various courses and studies sections. As such, future studies are warranted to replicate our findings in a more statistically robust way.

## Conclusion

The objective of our study was to *trans-*culturally adapt and test the factor structure, internal consistency/reliability, predictive validity, and sensitivity of the “Academic Flow Inventory” (4D 16). Our results showed an excellent internal consistency, a good temporal stability (test-retest), a good correlation matrix, good EFA factor loadings, and excellent CFA fit indices. Moreover, the sensitivity analysis investigated the impact of some variables (age, gender, kind of study) on the domain scores. All these findings enable us to conclude that this scale represents a good psychometric tool that can be used to quantitatively assess the academic flow level in a sample of students in the Arabic-speaking world. However, given the above-mentioned shortcomings, future studies are urgently needed, employing more heterogeneous samples from other Arabic-speaking countries and exploring the relationship of the academic flow with other psychological variables and constructs, especially those related to academic achievement, career expectations and future occupational employment.

## Data Availability Statement

All datasets generated for this study are included in the manuscript/supplementary files.

## Ethics Statement

All participants in our study were volunteers, taking part in the study in an anonymous and confidential way. They gave their written, informed consent to be part of the investigation and were carefully and extensively advised about the aim of the study. They were free to withdraw their participation at any moment of the investigation. The study protocol was in-depth reviewed and received the full approval by the ethical committee of the University of Sfax, Sfax, Tunisia. The patients/participants provided their written informed consent to participate in this study.

## Author Contributions

NC, NB, and FA conceived the experiment. NC, CA, HK, NB, and FA performed the experiment. NC, NG, NB, and FA analyzed the data. All authors wrote the manuscript.

## Conflict of Interest

The authors declare that the research was conducted in the absence of any commercial or financial relationships that could be construed as a potential conflict of interest.

## References

[B1] Archer G. E. B. Saltelli A. Sobol I. M. (1997). Sensitivity measures. anova – like techniques and the use of bootsrap. *J. Statis. Comput. Simul.* 58 99–120. 10.1080/00949659708811825

[B2] Borderie J. (2015). *La Quête du Team Flow Dans Les Jeux Vidéo Coopératifs : Apports Conceptuels et Méthodologiques.* Rennes: Université Rennes.

[B3] Borderie J. Michinov N. (2014). *Identifying Social Forms of Flow in Multi-User Videogames. Multiplayer 2: Compete - Cooperate – Communicate.* Münster: International conference on the social aspects of digital gaming.

[B4] Byrne B. M. (1998). *Multivariate Applications Book Series. Structural equation Modeling With LISREL, PRELIS, and SIMPLIS: Basic Concepts, Applications, and Programming.* Mahwah, NJ: Erlbaum Associates Publishers.

[B5] Carpentier J. Mageau G. A. Vallerand R. J. (2012). Ruminations and flow: why do people with a more harmonious passion experience higher well-being? *J. Happiness Stud.* 13 501–518. 10.1007/s10902-011-9276-4

[B6] Carvalho J. Chima F. O. (2014). Applications of structural equation modeling in social sciences research. *Am. Int. J. Contemp. Reasearch* 4 6–11.

[B7] Cortini M. Notarangelo E. Cardellicchio E. (2010). “Obscure future? a pilot study on university students’ career expectations,” in *Boundaryless Careers and Occupational Wellbeing*, eds Cortini M. Tanucci G. Morin E., (Berlin: Springer), 42–53.

[B8] Csikszentmihalyi M. (2004). *Vivre. La Psychologie Du Bonheur.* Paris: Robert Laffont.

[B9] Csikszentmihalyi M. Rathunde K. (1993). “The measurement of flow in everyday life: toward a theory of emergent motivation,” in *Current Theory and Research in Motivation Nebraska Symposium on Motivation, 1992: Developmental Perspectives on Motivation*, ed. Jacobs J. E., (Lincoln, NE: University of Nebraska Press), 57–97. 1340523

[B10] Debus M. E. Sonnentag S. Deutsch W. Nussbeck F. W. (2014). Making flow happen: the effects of being recovered on work-related flow between and within days. *J. Appl. Psychol.* 99 713–722. 10.1037/a0035881 24512028

[B11] Delle Fave A. Massimini F. (1988). “Modernization and the changing contexts of flow in work and leisure,” in *Optimal Experience*, eds Csikszentmihalyi M. Csikszentmihalyi I., (Cambridge: Cambridge University Press), 193–213. 10.1017/cbo9780511621956.012

[B12] Demontrond P. Gaudreau P. (2008). Le concept de "flow" ou "état psychologique optimal": etat de la question appliquée au sport. *Staps* 79 9–2.

[B13] Eisenberger R. Jones J. R. Stinglhamber F. Shanock L. Randall A. T. (2005). Flow experience at work: for high need achievers alone? *J. Organ. Behav.* 26 755–775. 10.1002/job.337

[B14] Engeser S. Baumann N. (2016). Fluctuation of flow and affect in everyday life: a second look at the paradox of work. *J. Happiness Stud.* 17 105–124. 10.1007/s10902-014-9586-4

[B15] Fong C. J. Zaleski D. J. Leach J. K. (2014). The challenge–skill balance and antecedents of flow: a meta-analytic investigation. *J. Positive Psychol.* 9760 1–22. 10.1080/17439760.2014.967799

[B16] Fournier J. Gaudreau P. Demontrond-Behr P. Visioli J. Forest J. Jackson S. A. (2007). French translation of the flow state scale-2: factor structure, cross-cultural invariance, and associations with goal attainment. *Psychol. Sport Exerc.* 8 897–916. 10.1016/j.psychsport.2006.07.007

[B17] Heutte J. Fenouillet F. (2010). “Propositions pour une mesure de l’expérience optimale (état de flow) en contexte éducatif,” in *Proceedings of the 26e Congrès International D’actualité de la Recherche en Éducation et en Formation (AREF 2010)*, (Genève).

[B18] Jackson S. A. Eklund R. C. (2002). Assessing flow in physical activity: the flow state scale-2 and dispositional flow scale-2. *J. Sport Exerc. Psychol.* 24 133–150. 10.1123/jsep.24.2.133

[B19] Jackson S. A. Marsh H. W. (1996). Development and validation of a scale to measure optimal experience: the flow state scale. *J. Sport Exerc. Psychol.* 18 17–35. 10.1123/jsep.18.1.17

[B20] Jackson S. A. Martin A. J. Eklund R. C. (2008). Long and short measures of flow: examining construct validity of the FSS -2, DFS -2, and new brief counterparts. *J. Sport Exerc. Psychol.* 30 561–587. 10.1123/jsep.30.5.561 18971512

[B21] Johnson D. W. Johnson R. T. (1989). *Cooperation and Competition: Theory and Research.* Edina, MN: Interaction Book Co.

[B22] Jöreskog K. Sörbom D. (1996a). *LISREL 8: User’s Reference Guide.* Chicago, IL: Scientific Software International Inc.

[B23] Jöreskog K. G. Sörbom D. (1996b). *LISREL8: User’s Reference Guide*, 2nd Edn Chicago, IL: Scientific Software International.

[B24] Kaiser H. F. (1958). The varimax criterion for analytic rotation in factor analysis. *Psychometrika* 23 187–200. 10.1007/bf02289233

[B25] Karageorghis C. I. Vlachopoulos S. Terry P. C. (2000). Latent variable modelling of the relationship between flow and exercise-induced feelings: an intuitive appraisal perspective. *Eur. Phys. Educ. Rev.* 6 230–248. 10.1177/1356336x000063002

[B26] Kulkarni A. Anderson W. Sanders M. A. Newbold J. Martin M. L. (2015). Manipulated flow reduces downstream defensiveness. *J. Positive Psychol.* 11 26–36. 10.1080/17439760.2015.1015157

[B27] Larson R. W. (2011). Positive development in a disorderly world. *J. Research Adolesc.* 21 317–334. 10.1111/j.1532-7795.2010.00707.x

[B28] MacCallum R. C. Browne M. W. Sugawara H. M. (1996). Power analysis and determination of sample size for covariance structure modeling. *Psychol. Methods* 1 130–149. 10.1037//1082-989x.1.2.130

[B29] Mäkikangas A. Bakker A. B. Aunola K. Demerouti E. (2010). Job resources and flow at work: modelling the relationship via latent growth curve and mixture model methodology. *J. Occup. Organ. Psychol.* 83 795–814. 10.1348/096317909x476333

[B30] Marsh H. W. Balla J. R. Mcdonald R. P. (1988). Goodness-of-fit indices in confirmatory factor analysis: the effect of sample size. *Psychol. Bull.* 102 391–410. 10.1037//0033-2909.103.3.391

[B31] Mayers P. (1978). *Flow in Adolescence and its Relation to School Experience.* Chicago, IL. Doctoral. dissertation.

[B32] Nakamura J. Csikszentmihalyi M. (2002). “The concept of flow,” in *Handbook of Positive Psychology*, eds Snyder C. Lopez S., (New York, NY: University Press), 89–105.

[B33] Quinn R. W. (2005). Flow in knowledge work: high performance experience in the design of national security technology. *Adm. Sci. Q.* 50 610–641. 10.2189/asqu.50.4.610

[B34] Rogatko T. P. (2009). The influence of flow on positive affect in college students. *J. Happiness Stud.* 10 133–148. 10.1007/s10902-007-9069-y

[B35] Roussel P. Durrieu F. Campoy E. El Akremi A. (2002). *Méthodes D’équations Structurelles : Recherches et Applications en Gestion.* Paris: Economica.

[B36] Salanova M. Bakker A. B. Llorens S. (2006). Flow at work: evidence for an upward spiral of personal and organizational resources. *J. Happiness Stud.* 7 1–22. 10.1007/s10902-005-8854-8

[B37] Satorra A. Bentler P. M. (1994). “Corrections to test statistics and standard errors in covariance structure analysis,” in *Latent Variables Analysis: Applications for Developmental Research*, eds von Eye A. Clogg C. C., (Thousand Oaks, CA: Sage Publications, Inc.), 399–419.

[B38] Schermelleh-Engel K. Moosbrugger H. Muller H. (2003). Evaluating the fit of structural equation models: tests of significance and descriptive goodness-of-fit measures. *Methods Psychol. Res.* 8 23–74.

[B39] Seligman M. E. P. Csikszentmihalyi M. (2000). Positive psychology: an introduction. *Am. Psychol.* 55 5–14. 10.1037/0003-066X.55.1.5 11392865

[B40] Walker C. J. (2010). Experiencing flow: is doing it together better than doing it alone? *J. Positive Psychol.* 5 3–11. 10.1080/17439760903271116

